# Florence Nightingale

**Published:** 1920-05-15

**Authors:** 


### Florence Nightingale.

The centenary of Florence Nightingale's birth was commemorated in Westminster Abbey on May 12 by a beautiful adaptation of the 3 p.m. Evensong. Deputations of nurses from various hospitals attended in uniform, and among the congregation were a number of representatives of hospital management and the medical profession. The anthem, Schubert's beautiful "Where Thou Dwellest," and the hymn, "The King of Love my Shepherd is," with the Ascension Day Collect, which came naturally into the order of the day, struck a welcome note of uplift and hope.

The Dean, Dr. Herbert Ryle, gave a short address on the text from Proverbs iii., "Many daughters have done wisely, but thou excellest them all." Three persons, he said, of the Victorian period in England had gloriously lessened human suffering: Simpson, Lister, and Florence Nightingale. When she died the honour of burial in the Abbey was offered, and later a place for her statue near that of Lord Shaftesbury. Sir Edward Cook's *Life* has given us, since 1913, a different view from that long current of a girl who gave up a brilliant life at the war's call, did miracles, and returned to the sheltered life of an invalid. We learned of a strong-souled, masterful woman whose fifty-four years' of life after the Crimea initiated reforms of barrack life, of Indian and English rural sanitation, besides her great work of transforming the nursing of the sick from a drudging labour to a great and noble profession. To have been crossed in love or proved unfit for other work ceased to be the natural qualification of a nurse. Training in skill, in knowledge, and in higher intellectual grasp for this she demanded till she got it, a standard of study and work against which some going through it may be tempted to rebel even now. She was a saint, but of no vapid or sentimental kind; she saw the hard head to be as much needed as the soft heart. She leaves the lesson that made life a worthy thing; it must not be frittered, but all its hours tend to one end—to God.

Many present must have been glad of the Dean's comment on the frivolous habit of contempt for the Victorian era and its figures. Pointing from corner to corner of the great church, he spoke of Tennyson and Browning, Beaconsfield, Gladstone, Macaulay, Shaftesbury, and others who rest there. Yes, it *is* a pity perhaps that the statue of Florence Nightingale does not stand there to represent the hallowing of the best strength of soul and mind and body in the great profession of nursing.

